# Enzyme Characterization of Pro-virulent SntA, a Cell Wall-Anchored Protein of *Streptococcus suis*, With Phosphodiesterase Activity on cyclic-di-AMP at a Level Suited to Limit the Innate Immune System

**DOI:** 10.3389/fmicb.2022.843068

**Published:** 2022-03-22

**Authors:** Alicia Cabezas, María Jesús Costas, José Canales, Rosa María Pinto, Joaquim Rui Rodrigues, João Meireles Ribeiro, José Carlos Cameselle

**Affiliations:** ^1^Grupo de Enzimología, Departamento de Bioquímica y Biología Molecular y Genética, Facultad de Medicina y Ciencias de la Salud, Universidad de Extremadura, Badajoz, Spain; ^2^Laboratório Associado Laboratory of Separation and Reaction Engineering-Laboratory of Catalysis and Materials (LSRE-LCM), Escola Superior de Tecnologia e Gestão, Instituto Politécnico de Leiria, Leiria, Portugal

**Keywords:** *Streptococcus suis*, virulence, SntA, 3′-nucleotidase, 2′,3′-cyclic phosphodiesterase, cyclic dinucleotide, c-di-AMP phosphodiesterase, type-I interferon response

## Abstract

*Streptococcus suis* and *Streptococcus agalactiae* evade the innate immune system of the infected host by mechanisms mediated by cell wall-anchored proteins: SntA and CdnP, respectively. The former has been reported to interfere with complement responses, and the latter dampens STING-dependent type-I interferon (IFN) response by hydrolysis of bacterial cyclic-di-AMP (c-di-AMP). Both proteins are homologous but, while CdnP has been studied as a phosphohydrolase, the enzyme activities of SntA have not been investigated. The core structure of SntA was expressed in *Escherichia coli* as a GST-tagged protein that, after affinity purification, was characterized as phosphohydrolase with a large series of substrates. This included 3′-nucleotides, 2′,3′-cyclic nucleotides, cyclic and linear dinucleotides, and a variety of phosphoanhydride or phosphodiester compounds, most of them previously considered as substrates of *E. coli* CpdB, a periplasmic protein homologous to SntA and CdnP. Catalytic efficiency was determined for each SntA substrate, either by dividing parameters *k*_*cat*_/*K*_*M*_ obtained from saturation curves or directly from initial rates at low substrate concentrations when saturation curves could not be obtained. SntA is concluded to act as phosphohydrolase on two groups of substrates with efficiencies higher or lower than ≈ 10^5^ M^–1^ s^–1^ (average value of the enzyme universe). The group with *k*_*cat*_/*K*_*M*_ ≥ 10^5^ M^–1^ s^–1^ (good substrates) includes 3′-nucleotides, 2′,3′-cyclic nucleotides, and linear and cyclic dinucleotides (notably c-di-AMP). Compounds showing efficiencies <10^4^ M^–1^ s^–1^ are considered poor substrates. Compared with CpdB, SntA is more efficient with its good substrates and less efficient with its poor substrates; therefore, the specificity of SntA is more restrictive. The efficiency of the SntA activity on c-di-AMP is comparable with the activity of CdnP that dampens type-I IFN response, suggesting that this virulence mechanism is also functional in *S. suis*. SntA modeling revealed that Y530 and Y633 form a sandwich with the nitrogen base of nucleotidic ligands in the substrate-binding site. Mutants Y530A-SntA, Y633A-SntA, and Y530A+Y633A-SntA were obtained and kinetically characterized. For orientation toward the catalytic site, one tyrosine is enough, although this may depend on the substrate being attacked. On the other hand, both tyrosines are required for the efficient binding of good SntA substrates.

## Introduction

Cell wall-anchored proteins are generally considered as pro-virulent factors of Gram-positive bacteria (Bergmann and Hammerschmidt, 2006; Bierne and Dramsi, 2012; Pietrocola et al., 2018; Soh et al., 2020). This is the case for SntA of *Streptococcus suis* and CdnP of *Streptococcus agalactiae*. Both proteins favor the virulence of the bacteria that encode them, although they have been proposed to act through different mechanisms. SntA is overexpressed under iron starvation (Li et al., 2009), and it is a heme-binding protein that favors iron acquisition from infected host reservoirs, also inhibiting host antioxidant protein AOP2 (Wan et al., 2017). Moreover, SntA interferes with the action of complement (Deng et al., 2018). Instead, CdnP interferes with the type-I IFN response of the infected host (Andrade et al., 2016).

**FIGURE 1:**
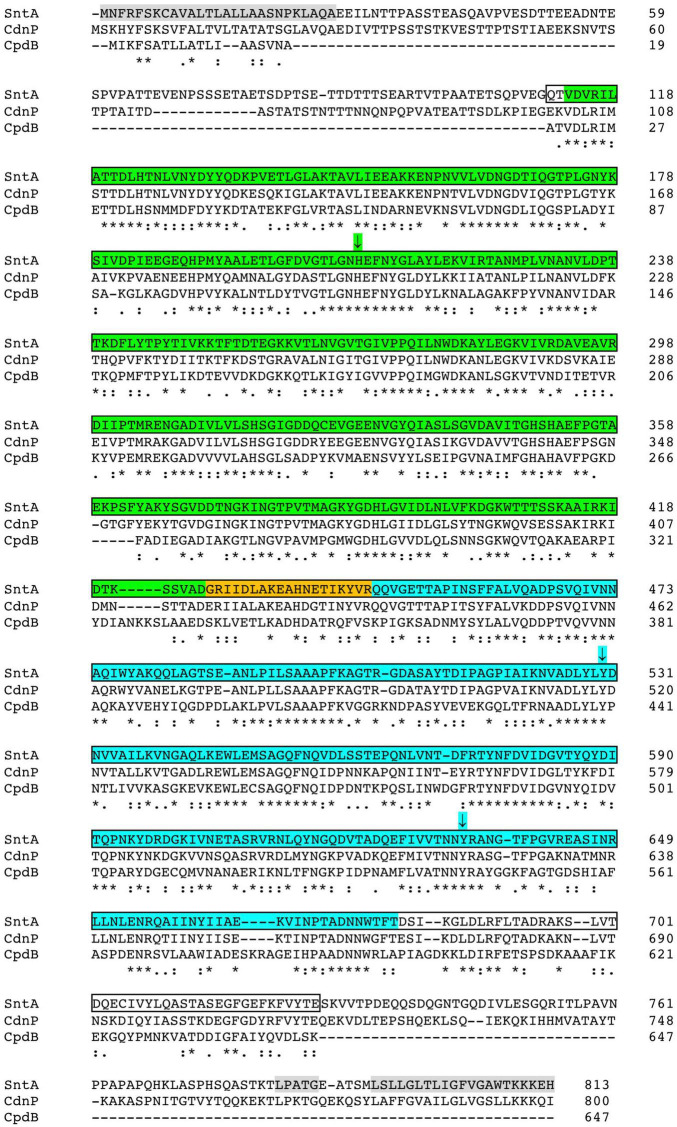
Sequence alignment of streptococcal SntA and CdnP with *Escherichia coli* CpdB. Sequences correspond to full protein precursors taken from GenBank accessions AYV64543 (SntA; this work), WP_000033934 (CdnP) (Andrade et al., 2016), and WP_000589409 (CpdB) (López-Villamizar et al., 2016). Alignment was prepared with Clustal Omega online (https://www.ebi.ac.uk/Tools/msa/clustalo/; accessed January 15, 2022). The following elements are indicated, from N to C end, on SntA precursor sequence: signal peptide (gray; amino acids 1–27) as predicted by SignalP 6.0 (Teufel et al., 2022); metallophos domain (green; amino acids 113–426); interdomain linker (orange; amino acids 427–446); 5_nucleotid_C domain (cyan; amino acids 447–680); and C-terminal sorting signal (gray) comprising a LPXTG motif (amino acids 783–787) and a hydrophobic domain with a positively charged tail (amino acids 792–813). SntA core used to run substrate specificity and kinetics experiments corresponds to amino acids 111–727 (shown within borders), which align well with mature CpdB. According to NCBI Blast, SntA and CdnP display ≈ 60% identity and ≈ 74% similarity to each other over 99% of their sequences. Each of streptococcal proteins displays ≈ 45% identity and ≈ 63% similarity to CpdB over 97% of CpdB sequence. (*) Fully conserved residue; (:) strongly conserved residues; (.) weakly conserved residues. Capital letters are standard for amino acids.

SntA and CdnP are structurally related to each other and to the periplasmic CpdB protein of *Escherichia coli* (López-Villamizar et al., 2016). Figure 1 shows a sequence alignment of the three proteins. They are synthesized with a removable N-terminal signal peptide (SP) for secretion, and they share metallophos (Pfam ID PF00149; also known as calcineurin-like phosphoesterase) and 5_nucleotid_C (PF02872) domains. This architecture is non-exclusively typical of 5′-nucleotidases like the archetypical UshA protein of *E. coli* (Knöfel and Sträter, 1999). Actually, CpdB is a highly efficient 3′-nucleotidase devoid of 5′-nucleotidase activity (López-Villamizar et al., 2016). In addition to the SP/metallophos/5_nucleotid_C architecture, the streptococcal SntA and CdnP, but not the much shorter CpdB, contain a C-terminal sorting signal with an LPXTG motif followed by a hydrophobic domain and a positively charged tail (Figure 1). This arrangement leads to a cleavage between the threonine and glycine residues of the LPXTG motif and to the covalent anchorage of the protein to mature peptidoglycan through the SrtA-mediated sorting pathway (Schneewind and Missiakas, 2012). An aspect shared by the three proteins considered is their access to the bacterial extracytoplasmic compartment, either from the periplasm of Gram-negative or the cell wall of Gram-positive bacteria.

Concerning enzyme activities, *E. coli* CpdB (López-Villamizar et al., 2016) and *S. agalactiae* CdnP (Andrade et al., 2016) have been independently characterized as phosphodiesterases of 2′,3′-cNMP and c-di-NMP. CpdB is also active as 3′-nucleotidase, as stated earlier, and as phosphodiesterase of linear dinucleotides, activities not tested in the case of CdnP but that can be inferred from the apparent direct conversion of 2′,3′-cNMP to adenosine, and of c-di-NMP to 5′-NMP. As far as we know, the possible enzyme activities of *S. suis* SntA have not been studied.

The enzyme activities of CpdB and CdnP have been related to the role of these proteins in host–pathogen interactions. On the one hand, there is evidence for a role in virulence of the *cpdB* gene of Gram-negative bacteria, including *Salmonella enterica* serovar Pullorum in an intraperitoneal chicken infection model (Liu et al., 2015), and avian pathogenic *E. coli* in an oral model of infection of specific pathogen-free chicken (Liu et al., 2017). In both cases, the deletion of the *cpdB* gene strongly diminished the long-term colonization of infected animals. On the other hand, the role of CdnP in diminishing the IFN response of mice infected with *S. agalactiae* has been demonstrated by comparing wild-type to bacteria bearing a *cdnP* mutant with an enzyme-inactivating point mutation (Andrade et al., 2016). These effects are difficult to explain in hydrolytic activity over 2′,3′-cNMP and 3′-nucleotides, but they can be rationalized by the extracytoplasmic hydrolysis of cyclic dinucleotides (Andrade et al., 2016; López-Villamizar et al., 2016), as these compounds are pathogen-associated molecular patterns (PAMP) that trigger innate immunity in the infected host (Woodward et al., 2010; Burdette et al., 2011; Parvatiyar et al., 2012; Danilchanka and Mekalanos, 2013; Sun et al., 2013; Wu et al., 2013; Dey and Bishai, 2014; Yang et al., 2014; Andrade et al., 2016; Devaux et al., 2018). In the case of *S. agalactiae*, it has been demonstrated that CdnP hydrolysis of pathogen-secreted cyclic-di-AMP (c-di-AMP) modulates STING-dependent type I IFN production and favors pathogen virulence in mice (Andrade et al., 2016). A similar case has been reported for another pro-virulent cyclic nucleotide phosphodiesterase of *Mycobacterium tuberculosis* encoded by the *Rv2837c* [or *cnpB* (Yang et al., 2014)] gene, which, although incidentally named also CdnP, like the *S. agalactiae* protein, is not a metallophos/5_nucleotid_C but a DHH-DHHA1 domain, a soluble protein with an unclear outward/inward orientation in *M. tuberculosis* membrane (Manikandan et al., 2014; He et al., 2016; Dey et al., 2017; Li, 2017). The bacterial stratagem of interfering with innate immunity by the hydrolysis of cyclic dinucleotides makes these enzymes interesting targets to develop novel therapeutic strategies in infectious diseases (Andrade et al., 2016; Dey et al., 2017; Li, 2017; Devaux et al., 2018; Eaglesham and Kranzusch, 2020; Karanja et al., 2021; López-Villamizar et al., 2021).

*Streptococcus suis* is an important swine and zoonotic pathogen, for which many virulence factors have been identified (Fulde and Valentin-Weigand, 2013; Goyette-Desjardins et al., 2014; Vötsch et al., 2018; Tram et al., 2021). The SntA protein has been reported to be involved in the virulence of *S. suis* by favoring iron acquisition, inhibiting host antioxidant protein AOP2 and inhibiting the complement system (Wan et al., 2017; Deng et al., 2018). However, it is known that c-di-AMP occurs in *S. suis*, and an intracellular cyclic dinucleotide phosphodiesterase GdpP can be related to virulence (Du et al., 2014). Given the structural relatedness of SntA to *E. coli* CpdB and *S. agalactiae* CdnP, the enzyme characterization of SntA was undertaken in the search for novel perspectives about this protein as a mediator of host–pathogen interaction.

## Materials and Methods

### Molecular Cloning of the *sntA* Gene

Genomic DNA of *S. suis* strain CCUG 7984 was obtained from the Colección Española de Cultivos Tipo (CECT, Valencia, Spain), and it was used to amplify the coding sequence of the complete SntA protein with primers designed using the *sntA* gene sequence with GenBank accession number AB066354 complement (4411.6852) (Osaki et al., 2002). The primers used were ATGAATTTTCGTTTCAGTAAGTGTGCCG (forward) and TGAGGCTTGAACAAGGGC (reverse). This primer pair encompasses the 2,442-nt open reading frames (ORFs) of the full SntA protein plus a 3′ extension of 44 nucleotides. The amplicon obtained with the Advantage cDNA polymerase mix (Clontech, available from Takara Bio Europe SAS, Saint-Germain-en-Laye, France), bearing 3′ A extensions, was directly used for ligation with T4 DNA ligase to the linearized pGEM-T Easy vector that contains 3′ T extensions (pGEM-T Easy Vector System I; Promega Biotech Ibérica S.L., Alcobendas, Spain). The ligation mixture was used to transform competent JM109 cells (Promega). White colonies were selected, and their plasmids (High Pure Plasmid Isolation Kit, Roche, available from Merck Life Sciences, Madrid, Spain) were analyzed by DNA sequencing from both ends of the T/A cloning site (Sp6 and T7 sequencing primers). One plasmid (pGEM-T-Easy-sntA), which contained the full coding sequence of SntA confirmed by double-strand sequencing (Servicio de Genómica, Instituto de Investigaciones Biomédicas Alberto Sols, Consejo Superior de Investigaciones Científicas, Universidad Autónoma, Madrid), was selected for further work. These sequence data were deposited in the GenBank database under accession number MH660457. The full 813 amino-acid translation of this sequence (accession AYV64543) contained five amino-acid differences relative to the translation of the sequence used for polymerase chain reaction design (accession BAB83969).

### Construction of a Plasmid Coding for the SntA Core

The core or central part of SntA contains the N-terminal metallophos and the C-terminal 5_nucleotid_C domains bound by an interdomain linker. Its construction was performed starting from plasmid pGEM-T-Easy-SntA that contains the ORF coding for the full SntA protein (2,442 nt), including a signal peptide, the core, and a C-terminal sorting signal with the LPATG sequence (LPXTG motif), followed by a hydrophobic domain and a positively charged tail (Figure 1). The sequence coding for the SntA core was amplified with primers CTGACCGGATCCCAAACTGTTGATG (forward) and ATCGAGTCGACTCATTCAGTGTAGAC (reverse). The forward primer contained the first 13 nucleotides of the core-coding sequence (nucleotides 331–343 of the full SntA ORF; underlined) preceded by a 5′ extension with a *Bam*HI site (GGATCC) and six extra nucleotides. The reverse primer contained the reverse complement of the last 12 nucleotides of the core-coding sequence (nucleotides 2,170–2,181 of the full SntA ORF; underlined) preceded by a 5′ extension with a stop codon, a *Sal*I site (GTCGAC), and five extra nucleotides. The expected 1,877-nt amplicon was digested with *Bam*HI and *Sal*I and directionally inserted between the corresponding sites of plasmid pGEX-6P-3 (GE Healthcare Life Sciences, available from VWR International Eurolab SLU, Llinars del Vallès, Spain). Competent JM109 cells were transformed with the ligation mixture. Ampicillin-resistant colonies were selected and analyzed by sequencing. One plasmid (pGEX-6P-3-sntA_core) that contained the full coding sequence of the SntA core, confirmed by double-strand sequencing, was used for expression and mutagenesis. The SntA core protein is 617 amino acids long, corresponding to amino acids 111–727 of the complete SntA protein (accession AYV64543). The numbering of the full protein is conserved for the core protein.

### Site-Directed Mutagenesis

Point mutants H209A, Y530A, and Y633A of the SntA core were prepared by the QuikChange protocol (Agilent Technologies Spain, S.L., Las Rozas, Madrid, Spain) with plasmid pGEX-6P-3-sntA_core as the template, using the following mutagenic primers, each combined with its reverse complement oligonucleotide: H209A, CGGCACACTTGGCAACGCAGAATTTAACTACGGCCTTGC C; Y530A, CGTAGCAGACCTTTACCTCGCCGACAATGTTGT AGCCATC; Y633A, CATCGTGGTGACCAACAACGCCCGTGC AAATGGTACTTTC. This provided plasmids pGEX-6P -3-H209A-sntA_core, pGEX-6P-3-Y530A-sntA_core, and pGEX -6P-3-Y633A-sntA_core. The double mutant Y530A + Y633A was prepared using pGEX-6P-3-Y530A-sntA_core as the template with the same mutagenic primer pair used to prepare the Y633A mutant, which provided plasmid pGEX-6P-3-Y530A+Y633A-sntA_core. The correctness of all the mutated plasmids was confirmed by double-strand sequencing of the resulting full ORF.

### Expression of Recombinant Proteins

The constructs bearing the sequences coding for the SntA core and its mutants contain the cloned coding sequences in frame with those of the PreScission protease cut sequence and the glutathione S-transferase (GST) label. The recombinant proteins can be expressed from the isopropyl ß-D-1-thiogalactopyranoside-inducible tac promoter of the pGEX-6P-3 vector as GST fusions that can be cut with PreScission protease (GE Healthcare Life Sciences, available from VWR International Eurolab SLU, Llinars del Vallès, Spain) to yield the desired SntA protein with a GPLGS N-terminal extension. The expression and purification of the recombinant proteins were performed as described (López-Villamizar et al., 2016). In brief: each expression construct was used to transform BL-21 cells; ampicillin-resistant colonies were selected, cultured in suspension, induced by isopropyl ß-D-1-thiogalactopyranoside, and collected by centrifugation. The cells were resuspended in the presence of a protease inhibitor cocktail, lysed by sonication, and the lysis supernatant was taken for purification of the recombinant proteins. Purification was accomplished by affinity chromatography on GSH-Sepharose (GE Healthcare Life Sciences, available from VWR International Eurolab SLU, Llinars del Vallès, Spain), followed by separation from the GST label by specific proteolysis with PreScission.

### Enzyme Activity Assays and Estimation of Kinetic Parameters

All the enzyme activities catalyze the hydrolysis of either phosphomonoester, phosphodiester, or phosphoanhydride linkages. The standard reaction mixtures contained 50-mM Tris–HCl, pH 7.5 at 37°C, 2-mM MnCl_2_, 0.1 mg ml^–1^ bovine serum albumin, diverse concentrations of substrate and recombinant enzyme. The hydrolysis of nucleoside-mono-, di-, and triphosphates and 4-NPhP were assayed, measuring colorimetrically the amount of phosphate directly formed as a product. The hydrolysis of cyclic mononucleotides, dinucleoside-oligophosphates, nucleoside-diphosphate sugar or choline, and bis-4NPhP were similarly assayed but in the presence of an excess of alkaline phosphatase to liberate phosphate from the reaction products. The hydrolysis of linear or cyclic dinucleotides was assayed by high-performance liquid chromatography. Details of inorganic phosphate assay and liquid chromatography methods were as described (López-Villamizar et al., 2016). All the assays were run at 37°C, under conditions of linearity with respect to incubation time and enzyme amount. Controls without enzyme and/or substrate were run in parallel and their results subtracted from those obtained in full reaction mixtures as required.

The kinetic parameters *k*_*cat*_ and *K*_*M*_ were estimated from saturation experiments where initial rates were measured at diverse substrate concentrations. The Michaelis–Menten equation was adjusted to the experimental data points by non-linear regression using the Solver tool of Microsoft Excel 2011 for the Mac. *V*_*max*_ (to be converted to *k*_*cat*_) and *K*_*M*_ were allowed to fluctuate during the adjustment. The catalytic efficiency equals the quotient *k*_*cat*_/*K*_*M*_, but when saturation plots could not be obtained, this parameter was derived from initial rate data obtained at substrate concentrations much lower than the *K*_*M*_, i.e., when the initial rate is practically proportional to substrate concentration and most part of the enzyme is in free form. Under these conditions, *k*_*cat*_/*K*_*M*_ = *v*/([E][S]), [E] being the total concentration of enzyme (Fehrst, 1998).

## Results and Discussion

For characterization of SntA, we chose to construct and express the core or central part of the protein (amino acids 111–727) because it aligns well with mature *E. coli* CpdB (Figure 1), earlier characterized for specificity and kinetics (López-Villamizar et al., 2016). The core SntA protein includes the metallophos and 5-nucleotid_C domains that contain, respectively, the catalytic site and the substrate-binding pocket of CpdB (López-Villamizar et al., 2021). Amino acids 1–27 form the signal sequence, which would be absent from the mature protein. The amino-acid stretch 28–110, not included in the expressed protein, is likely disordered, according to its predicted lack of secondary structure (Drozdetskiy et al., 2015). Moreover, it is far from the active site, and it does not affect the folding of the core protein domains, according to preliminary models obtained in the Phyre2 web portal (Kelley et al., 2015) for full SntA and the semi-mature protein (devoid of signal peptide but not yet processed at the LPXTG motif). Therefore, it is unlikely that amino acids 28–110 play a specific role in enzyme activity. The C-terminal region (amino acids 728–813), also not included in the expressed protein, is involved in anchorage to the cell wall, possibly forming a molecular stem after removal of amino acids 787–813 by processing at the LPXTG motif.

### Substrate Specificity of *Streptococcus suis* SntA

A large study of substrate specificity of the SntA core (in the following just referred to as SntA) is summarized in Table 1, including the assay of kinetic parameters *k*_*cat*_, *K*_*M*_, and *k*_*cat*_/*K*_*M*_. The set of substrates considered includes 3′-nucleotides, 2′,3′-cyclic nucleotides, cyclic and linear dinucleotides, and a variety of phosphoanhydride or phosphodiester compounds, most of them previously considered in a study of the substrate specificity of *E. coli* CpdB (López-Villamizar et al., 2016). For this kinetics and specificity study, Mn^2+^ was chosen as the activating cation because this was also the metal of choice in the published studies of the structurally related CpdB and CdnP proteins (Andrade et al., 2016; López-Villamizar et al., 2016) as well as in other c-di-NMP phosphodiesterases (Du et al., 2014; He et al., 2016). In addition, it is known that Mn^2+^ is important for *S. suis* pathogenesis (Wichgers Schreur et al., 2011; Xu et al., 2017).

**TABLE 1:** Kinetic parameters of SntA.

	*k*_*cat*_ (s^–1^)	*K*_*m*_ (μM)	*k*_*cat*_/*K*_*m*_ (M^–1^ s^–1^)
3′-GMP	666.55 ± 58.31	4.05 ± 1.01	1.65E+08
3′-AMP	323.76 ± 13.34	2.62 ± 0.28	1.23E+08
2′,3′-cAMP	290.51 ± 9.13	3.97 ± 1.62	7.31E+07
3′-UMP	573.53 ± 17.09	20.42 ± 2.90	2.81E+07
2′,3′-cGMP	318.30 ± 15.40	16.50 ± 0.60	1.93E+07
2′,3′-cCMP	373.45 ± 10.82	36.75 ± 3.45	1.02E+07
pApA	19.33 ± 3.51	2.86 ± 0.24	6.76E+06
2′,3′-cUMP	748.84 ± 32.83	208.04 ± 21.40	3.60E+06
bis-4NPhP	273.66 ± 13.64	406.57 ± 71.02	6.73E+05
c-di-AMP	1.65 ± 0.43	2.66 ± 1.29	6.20E+05
pGpG	0.76 ± 0.13	1.99 ± 0.67	3.83E+05
3′,3′-cGAMP	0.28 ± 0.05	1.00 ± 0.33	2.82E+05
c-di-GMP	0.06 ± 0.00	0.77 ± 0.48	8.07E+04
ATP	0.85 ± 0.15	92.70 ± 31.97	9.13E+03
Ap4A	0.29 ± 0.02	40.52 ± 1.12	7.09E+03
Ap3A	0.41 ± 0.03	62.45 ± 12.91	6.63E+03
4-NPhP	4.98 ± 0.24	2,680.57± 118.83	1.86E+03
ADP	0.17 ± 0.00	108.50 ± 9.66	1.57E+03
2′,3′-cGAMP	0.01 ± 0.00	20.51 ± 7.06	6.18E+02
ADP-ribose	0.08 ± 0.00	154.98 ± 19.06	5.30E+02
3′,5′-cAMP	Nd	Nd	7.87E+01
CDP-choline	0.26 ± 0.03	3,650.32 ± 563.54	7.19E+01
ADP-glucose	Nd	Nd	2.67E+01
UDP-glucose	Nd	Nd	1.73E+01
5′-AMP*	Nd	Nd	Nd
2′-AMP*	Nd	Nd	Nd

*k_cat_ and K_M_ values were calculated from saturation curves obtained at different concentrations of substrates.*

*They are shown as mean values ± standard deviations of three experiments.*

*Catalytic efficiencies were calculated by dividing k_cat_/K_M_ or, when these parameters were not available, by the procedure described in the section “Materials and Methods.”*

*Nd, not determined.*

*In assays at a fixed 200-μM concentration, activities on 5′-AMP and 2′-AMP represented <0.0005% of activity on 3′-GMP and <25% of activity on CDP-choline.

From Table 1, SntA emerges as a broad-specificity phosphohydrolase showing marked selectivity for some substrates vs. the others, as reflected in the 10^7^-fold span of catalytic efficiencies (*k*_*cat*_/*K*_*M*_), from approximately 20 M^–1^ s^–1^ up to near 2 × 10^8^ M^–1^ s^–1^. The best substrates are purine 3′-nucleotides with *k*_*cat*_/*K*_*M*_ > 10^8^ M^–1^ s^–1^, the theoretical maximum of catalytic activity shown by diffusion-controlled encounters of enzyme and substrate (Fehrst, 1998; Bar-Even et al., 2015). Next, come pyrimidine 3′-nucleotides, 2′,3′-cyclic nucleotides, and the linear dinucleotide pApA with *k*_*cat*_/*K*_*M*_ 10^6^–10^8^ M^–1^ s^–1^, followed by 3′,3′-cyclic dinucleotides, the linear dinucleotide pGpG, and the artificial phosphodiester bis-4NPhP with *k*_*cat*_/*K*_*M*_ 10^5^–10^6^ M^–1^ s^–1^.

Although catalytic efficiencies of 10^5^–10^6^ M^–1^ s^–1^ are 10^2^–10^3^-fold lower than those shown by SntA for purine 3′-nucleotides, they are not negligible in absolute terms, as the catalytic efficiency of the average enzyme is ≈ 10^5^ M^–1^ s^–1^ (Bar-Even et al., 2011). In this article, we shall consider good or poor substrates, respectively, those displaying catalytic efficiencies ≥10^5^ M^–1^ s^–1^ or <10^4^ M^–1^ s^–1^ (Table 1).

Using this somewhat arbitrary criterion, c-di-AMP falls among the good substrates of SntA. However, this is not the only reason to consider that the hydrolysis of c-di-AMP by *S. suis* SntA can be physiologically significant. Comparing SntA with *S. agalactiae* CdnP (Andrade et al., 2016), their catalytic efficiencies for the hydrolysis of c-di-AMP are rather similar. SntA shows a ≈ 10-fold lower *k*_*cat*_ and a ≈fivefold lower *K*_*M*_ than CdnP, so that SntA *k*_*cat*_/*K*_*M*_ (6.2 × 10^5^ M^–1^ s^–1^) is half of that of CdnP (1.2 × 10^6^ M^–1^ s^–1^). With this level of CdnP activity over c-di-AMP, *S. agalactiae* degrades the compound extracellularly and dampens STING-dependent type I IFN responses in mice. It follows that SntA may have similar significance as a virulence factor in *S. suis*, besides its other reported pro-virulent effects (Wan et al., 2017; Deng et al., 2018).

### Comparison of the Substrate Specificity of *Streptococcus suis* SntA to *Escherichia coli* CpdB

The availability of large sets of substrate data for *S. suis* SntA (Table 1) and for *E. coli* CpdB (López-Villamizar et al., 2016) provides the opportunity to analyze in detail the substrate preferences of these related enzymes in Gram-positive and Gram-negative bacteria. First of all, concerning their activities on cyclic dinucleotides, the catalytic efficiencies of SntA for the hydrolysis of c-di-AMP and c-di-GMP are higher than those of CpdB. In this comparison, the behavior of c-di-AMP is especially interesting, as Gram-positives, such as *S. suis*, and Gram-negatives, such as *E. coli*, differ in their production and role.

C-di-AMP is a signaling molecule widely produced by Gram-positives, where it plays in many aspects of physiology as recently reviewed (Commichau et al., 2019; Yin et al., 2020; Mudgal et al., 2021). It is known to be involved in the control of osmotic pressure (Commichau and Stülke, 2018), central metabolism (Sureka et al., 2014), genome integrity (Witte et al., 2008), biofilm formation (Peng et al., 2016), and sporulation (Oppenheimer-Shaanan et al., 2011). These are bacterial functions that respond to the intracellular turnover of c-di-AMP. In addition, and depending on its extracellular turnover during infection, c-di-AMP is recognized as a PAMP that activates host immune response (Woodward et al., 2010; Parvatiyar et al., 2012; Danilchanka and Mekalanos, 2013; Yang et al., 2014; Andrade et al., 2016; Devaux et al., 2018). The production and effects of c-di-AMP in Gram-negatives are also important but less widespread (Barker et al., 2013; López-Villamizar et al., 2016; Rubin et al., 2018).

**FIGURE 2:**
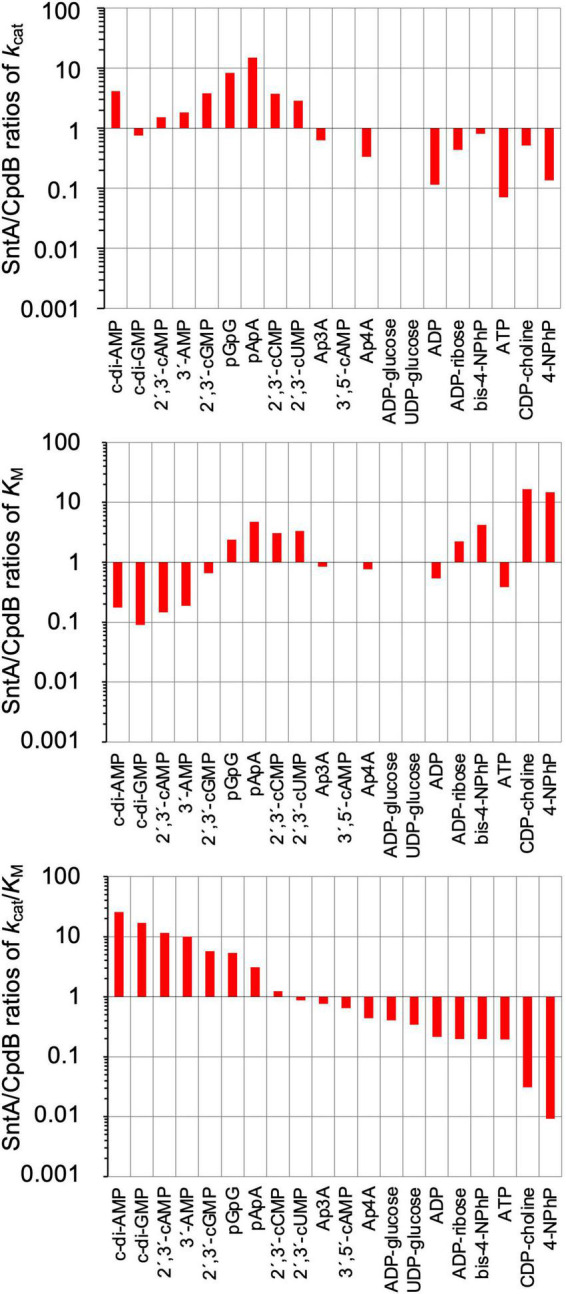
Comparison of substrate specificities of *Streptococcus suis* SntA and *Escherichia coli* CpdB. Kinetic parameters shown in Table 1 are divided by kinetic parameters of CpdB (López-Villamizar et al., 2016). Results are shown in log scale. In all panels, substrates are ordered by decreasing SntA/CpdB ratios of catalytic efficiency *k*_*cat*_/*K*_*M*_. Two groups of substrates can be distinguished depending on whether SntA/CpdB ratio of efficiency was clearly higher or lower than unity (lower panel).

The detailed comparison between SntA and CpdB is presented in Figure 2, in the form of SntA/CpdB ratios of *k*_*cat*_, *K*_*M*_, and *k*_*cat*_/*K*_*M*_. In the three panels, the substrates are shown left to right in decreasing order of their SntA/CpdB ratios of catalytic efficiencies. They fall into two groups: substrates for which SntA showed higher catalytic efficiency than CpdB and *vice versa* (Figure 2, lower panel).

The first group of substrates is formed by those with SntA/CpdB ratios of catalytic efficiencies clearly above unity, including c-di-AMP, c-di-GMP, 2′,3′-cAMP, 3′-AMP, 2′,3′-cGMP, pGpG, and pApA. All of them are good SntA substrates in absolute terms with *k*_*cat*_/*K*_*M*_ ≥ 10^5^ M^–1^ s^–1^ (Table 1). Noteworthy, c-di-AMP was a 25-fold better substrate for SntA than for CpdB, and it was the only one for which the higher SntA efficiency was the consequence of a large *k*_*cat*_ ratio concomitant with a small *K*_*M*_ ratio. For the other substrates in this group, the high SntA/CpdB efficiency ratios depend either on a small ratio of *K*_*M*_ values with similar *k*_*cat*_ values for both enzymes (c-di-GMP, 2′,3′-cAMP, and 3′-AMP) or on a large ratio of *k*_*cat*_ values with similar *K*_*M*_ values for both enzymes (2′,3′-cGMP) or even with relatively large ratios of *K*_*M*_ values (pGpG and pApA).

The second group of substrates is formed by those with SntA/CpdB ratios of catalytic efficiencies clearly below unity. With the exception of the artificial phosphodiester bis-4NPhP, all these compounds are not good SntA substrates according to their *k*_*cat*_/*K*_*M*_ < 10^4^ M^–1^ s^–1^ (Table 1).

The differences between SntA and CpdB concerning these two groups of substrates depict SntA as an enzyme with a more stringent specificity than CpdB, as the good substrates of SntA display a gain of catalytic efficiency relative to CpdB, whereas the poor substrates of SntA display losses of catalytic efficiency relative to CpdB (Figure 2, lower panel). Speculatively, this could be due to subtle differences in the substrate-binding center and/or in the relative positions of the substrate-binding pocket and the catalytic site of both enzymes during a possible hinge-bending rotation of the 5_nucleotid_C domain (see later).

### Model of SntA Structure and Position of Amino Acids Relevant to Substrate Binding: Effects of Mutations on SntA Kinetics

**FIGURE 3:**
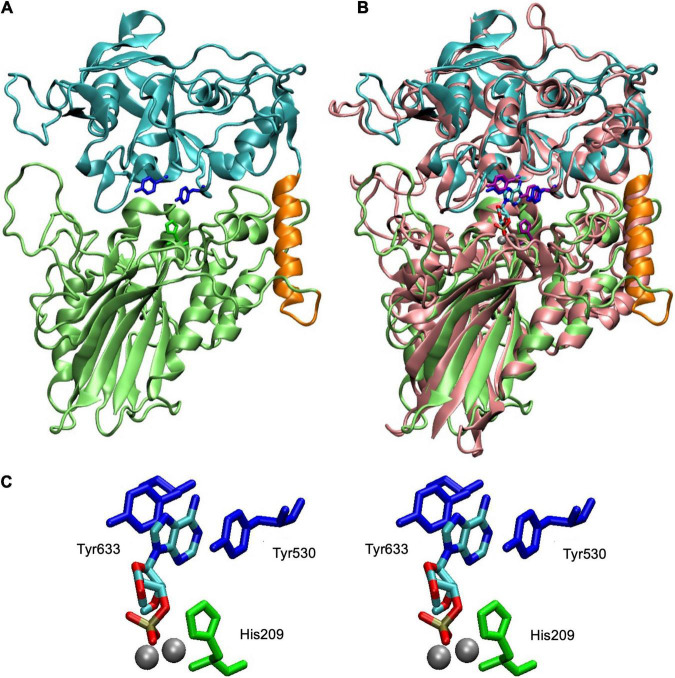
Structure of SntA and fit of 3′-AMP into active site. **(A)** Homology model covering residues 113–680 of complete SntA protein (GenBank accession AYV64543). Model was built in ORION server (https://www.dsimb.inserm.fr/ORION/) with Modeler (Sali and Blundell, 1993; Ghouzam et al., 2015, 2016) using Protein Data Bank (PDB) ID 3QFK structure as template and includes metallophos (113–426; lime) and 5_nucleotid_C (447–680; cyan) domains and linker region (427–446; orange). Relevant amino acids are highlighted: His209 (in metallophos domain; green), Tyr530 and Tyr633 (both in 5_nucleotid_C domain; blue). **(B)** Structural alignment of SntA model (panel A) with an *Escherichia coli* CpdB model with 3′-AMP docked and two Mg ions in the dinuclear metallic center (López-Villamizar et al., 2021). Alignment was generated with MultiSeq plugin (Roberts et al., 2006) of Visual Molecular Dynamics program (Humphrey et al., 1996). CpdB amino acids equivalent to those highlighted in panel **(A)** are shown (His117, Tyr440, and Tyr544, in CpdB; purple); metals ions are in silver; 3′-AMP is colored by chemical element. **(C)** Stereogram of SntA relevant amino acids displaying their relationship with 3′-AMP and metal ions (imported from CpdB model). Tyr530 and Tyr633 are amino acids mutated to alanine (single and double mutants) discussed in detail in this paper. His209 is conserved in metallophos domains of many proteins and is needed for catalysis, as known, for instance, from study of CpdB (López-Villamizar et al., 2021). SntA mutant H209A confirmed this role. Figure was prepared with Visual Molecular Dynamics program.

A theoretical model of the core of SntA (Figure 3), such as the full, mature CpdB protein (López-Villamizar et al., 2021), displays a two-domain structure with metallophos and 5_nucleotid_C domains bound by a ≈ 20 amino acid linker. In the case of CpdB, the protein has been dissected, and the two domains have been expressed separately and have been shown to play different roles in the catalytic cycle: a substrate-binding site occurs in the 5_nucleotid_C domain, whereas the catalytic site occurs in the metallophos domain (López-Villamizar et al., 2021). This is also the pattern present in the structurally related 5′-nucleotidase UshA of *E. coli*, which during its catalytic cycle, after the binding of substrate to an open conformation of the enzyme, undergoes a hinge-bending rotation of the 5_nucleotid_C domain that brings substrate to the catalytic site in the metallophos domain (Knöfel and Sträter, 1999, 2001; Krug et al., 2013). This kind of rotation has been postulated to happen during the catalytic cycle of CpdB (López-Villamizar et al., 2021), and it could also happen in the case of SntA.

**FIGURE 4:**
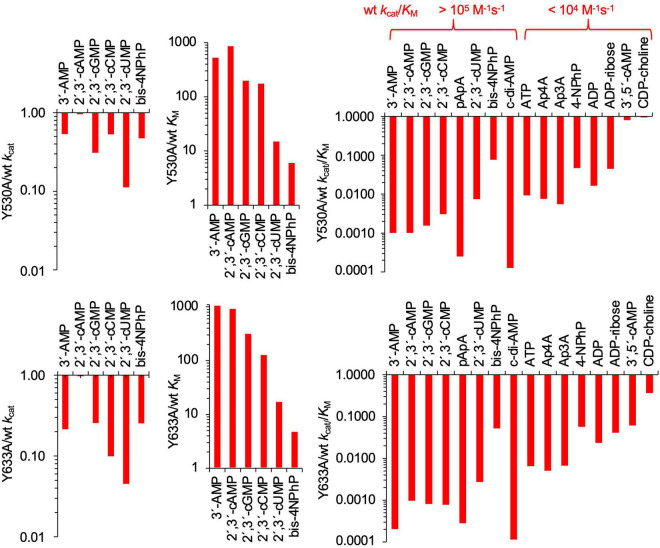
Effects of Y530A and Y633A mutations on kinetic parameters of SntA. Absolute values of *k*_*cat*_, *K*_*M*_, and *k*_*cat*_/*K*_*M*_ are shown in Tables 2, 3. Panels show in log scale ratios of these data vs. wild-type parameters of Table 1. The indication made in upper end of right panels classifies substrates into two groups depending on their wild-type catalytic efficiencies, here called good and poor SntA substrates.

In the substrate-binding site of SntA, two tyrosine residues (Y530 and Y633) are positioned to form a sandwich with, e.g., the purine ring of 3′-AMP (Figure 3). To explore the relevance of this interaction in the substrate specificity of SntA, the mutants Y530A and Y633A were constructed. They were tested for enzyme activity with a selection of substrates, including several that belong to the good-substrate type (*k*_*cat*_/*K*_*M*_ ≥ 10^5^ M^–1^ s^–1^) and others that belong to the poor-substrate type (*k*_*cat*_/*K*_*M*_ < 10^4^ M^–1^ s^–1^). The kinetic results obtained with the mutants are shown in Tables 2, 3. Comparisons with the wild-type enzyme are made in Figure 4.

**TABLE 2:** Kinetic parameters of Y530A-SntA.

	*k*_*cat*_ (s^–1^)	*K*_*m*_ (μM)	*k*_*cat*_/*K*_*m*_ (M^–1^ s^–1^)
3′-AMP	172.85 ± 9.57	1,379.38 ± 145.11	1.25E+05
2′,3′-cAMP	277.34 ± 59.26	3,418.12 ± 790.15	8.11E+04
2′,3′-cGMP	98.02 ± 14.99	3,245.54 ± 503.74	3.02E+04
2′,3′-cCMP	197.89 ± 33.86	6,440.94 ± 1,528.91	3.07E+04
pApA	Nd	Nd	1.68E+03
2′,3′-cUMP	83.75 ± 3.32	3,124.20 ± 292.86	2.68E+04
bis-4NPhP	129.66 ± 9.80	2,441.77 ± 211.17	5.31E+04
c-di-AMP	Nd	Nd	8.53E+01
ATP	Nd	Nd	8.93E+01
Ap4A	Nd	Nd	5.38E+01
Ap3A	Nd	Nd	3.74E+01
4-NPhP	Nd	Nd	8.75E+01
ADP	Nd	Nd	2.60E+01
ADP-ribose	Nd	Nd	2.40E+01
3′,5′-cAMP	Nd	Nd	6.29E+01
CDP-choline	Nd	Nd	6.79E+01

*k_cat_ and K_M_ values were calculated from saturation curves obtained at different concentrations of substrates.*

*They are shown as mean values ± standard deviations of three experiments.*

*Catalytic efficiencies were calculated by dividing k_cat_/K_M_ or, when these parameters were not available, by the procedure described in the section “Materials and Methods.”*

*Nd, not determined.*

**TABLE 3:** Kinetic parameters of Y633A-SntA.

	*k*_*cat*_ (s^–1^)	*K*_*m*_ (μM)	*k*_*cat*_/*K*_*m*_ (M^–1^ s^–1^)
3′-AMP	69.13 ± 3.50	2,769.37 ± 335.61	2.50E+04
2′,3′-cAMP	281.98 ± 27.76	3,625.43 ± 184.72	7.78E+04
2′,3′-cGMP	81.41 ± 21.53	5,190.24 ± 1,488.46	1.57E+04
2′,3′-cCMP	37.13 ± 10.29	4,704.97 ± 1,234.16	7.89E+03
pApA	Nd	Nd	1.91E+03
2′,3′-cUMP	33.90 ± 15.37	3,583.27 ± 2,010.97	9.46E+03
bis-4NPhP	69.18 ± 8.75	1,923.44 ± 208.29	3.60E+04
c-di-AMP	Nd	Nd	7.78E+01
ATP	Nd	Nd	6.18E+01
Ap4A	Nd	Nd	3.59E+01
Ap3A	Nd	Nd	4.55E+01
4-NPhP	Nd	Nd	1.06E+02
ADP	Nd	Nd	3.69E+01
ADP-ribose	Nd	Nd	2.20E+01
3′,5′-cAMP	Nd	Nd	4.80E+00
CDP-choline	Nd	Nd	2,65E+01

*k_cat_ and K_M_ values were calculated from saturation curves obtained at different concentrations of substrates.*

*They are shown as mean values ± standard deviations of three experiments.*

*Catalytic efficiencies were calculated by dividing k_cat_/K_M_ or, when these parameters were not available, by the procedure described in the section “Materials and Methods.”*

*Nd, not determined.*

For the most part, Y530A and Y633A substitutions had similar effects, although, for several substrates, Y633A showed a somewhat stronger effect in lowering the *k*_*cat*_. Both mutations strongly diminished the catalytic efficiencies *k*_*cat*_/*K*_*M*_ with all the tested substrates except with the very minor CDP-choline and, in the case of Y530A, with 3′,5′-cAMP (Figure 4, right-hand panels). In the few cases where *k*_*cat*_ and *K*_*M*_ could be assayed, the loss of efficiency was related to changes of *K*_*M*_, either a strong increase of 100–1,000-fold (3′-AMP, 2′,3′-cAMP, 2′,3′-cGMP, and 2′,3′-cCMP) or a moderate 3–12-fold increase (2′,3′-cUMP and bis-4NPhP) (Figure 4, central panels). Moderate 5–10-fold decreases of *k*_*cat*_ were also observed, except for 2′,3′-cAMP, which showed no change of *k*_*cat*_ with either mutation (Figure 4, left panels). For many other substrates, only direct estimations of *k*_*cat*_/*K*_*M*_ were possible, so they are shown only in Figure 4 right panels. Interestingly, the two groups of SntA substrates mentioned earlier, defined as good or poor according to their wild-type catalytic efficiencies, manifested quantitative differences in their responses to Y530A and Y633A substitutions. For the group of natural substrates (i.e., the artificial substrate bis-4NPhP excluded) that belong to the good-substrate type (*k*_*cat*_/*K*_*M*_ ≥ 10^5^ M^–1^ s^–1^), the mutant/wild type ratios of *k*_*cat*_/*K*_*M*_ ranged ≈ 0.0001–0.01, whereas for the group of substrates that belong to the poor-substrate type (*k*_*cat*_/*K*_*M*_ < 10^4^ M^–1^ s^–1^), the mutant/wild type ratios of *k*_*cat*_/*K*_*M*_ ranged ≈ 0.01–1 (Figure 4, right panels).

**FIGURE 5:**
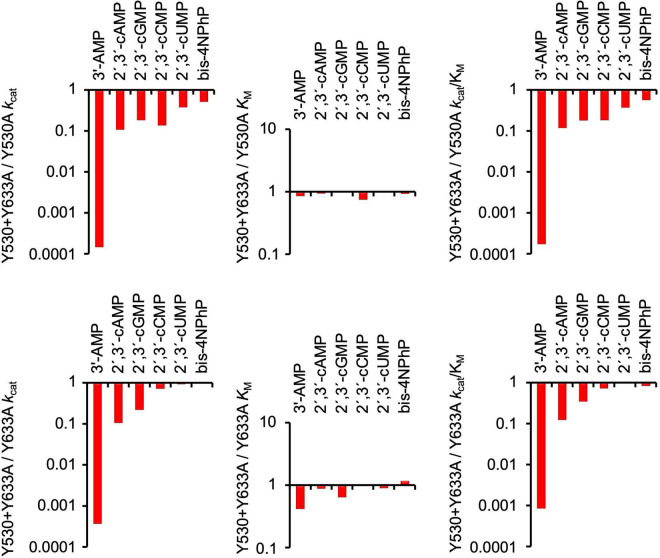
Comparison of effects of double SntA mutant Y530A + Y633A with single mutants Y530A and Y633A. Absolute values of *k*_*cat*_, *K*_*M*_, and *k*_*cat*_/*K*_*M*_ for double mutant are shown in Table 4. Panels show in log scale ratios of these data vs. parameters of single mutants (Tables 2, 3).

The kinetic study of Y530A-SntA and Y633A-SntA supports the role of the tyrosine residues in substrate binding, forming a sandwich with the nitrogen base as depicted in Figure 3. To get further insight into this matter, the double SntA mutant Y530A + Y633A was also constructed, and several good substrates were assayed: 3′-AMP, 2′,3′-cAMP, 2′,3′-cGMP, 2′,3′-cCMP, 2′,3′-cUMP, and bis-4NPhP. The kinetic results obtained with the double mutant are shown in Table 4, and their comparison with the single mutants is made in Figure 5. This comparison allows measuring the effect of eliminating the second tyrosine after having one of them removed.

**TABLE 4:** Kinetic parameters of double mutant Y530A+Y633A-SntA.

	*k*_*cat*_ (s^–1^)	*K*_*m*_ (μM)	*k*_*cat*_/*K*_*m*_ (M^–1^ s^–1^)
3′-AMP	0.025 ± 0.002	1,162.44 ± 84.76	2.16E+01
2′,3′-cAMP	29.33 ± 4.09	3,183.95 ± 926.67	9.21E+03
2′,3′-cGMP	17.65 ± 3.19	3,313.34 ± 446.14	5.33E+03
2′,3′-cCMP	26.39 ± 7.21	4,785.63 ± 1,562.63	5.51E+03
2′,3′-cUMP	30.95 ± 5.04	3,189.01 ± 607.81	9.71E+03
bis-4NPhP	65.85 ± 7.78	2,239.29 ± 327.54	2.94E+04

*k_cat_ and K_M_ values were calculated from saturation curves obtained at different concentrations of substrates.*

*They are shown as mean values ± standard deviations of three experiments.*

*Catalytic efficiencies were calculated by dividing k_cat_/K_M_.*

Among the substrates tested with the double mutant, 3′-AMP was the only one that displayed strong differences of *k*_*cat*_ between the double and the single mutants. Although the first tyrosine removal caused only a moderate decrease of *k*_*cat*_ with 3′-AMP (Figure 4, left panels), the removal of the second tyrosine caused an additional 10^3^–10^4^-fold decrease of *k*_*cat*_ (Figure 5, left panels). This indicates that for the correct orientation of 3′-AMP toward the catalytic site, one of the two tyrosines (any of them) is enough, as the drastic *k*_*cat*_ decrease occurred only after removal of the second tyrosine (i.e., when both were eliminated). Similar behavior was displayed by 2′,3′-cAMP but with lesser changes of *k*_*cat*_. Indeed, the removal of one tyrosine did not affect *k*_*cat*_ (Figure 4, left panels), and only after removal of the second tyrosine that a moderate decrease was observed (Figure 5, left panels). With the other substrates tested, the results were less clear-cut: both the removal of the first tyrosine (Figure 4, left panels) and of the second one (Figure 5, left panels) evoked moderate-to-null changes of *k*_*cat*_ to different degrees depending on the substrate. In these cases, both tyrosines affect substrate orientation in a small but additive fashion.

In contrast, in binding to the substrate pocket, the removal of the first tyrosine (any of them) caused strong-to-moderate increases of *K*_*M*_ for all the substrates tested (Figure 4, central panels), which were not further increased by the removal of the second tyrosine (Figure 5, central panels). This indicates that the elimination of just one tyrosine strongly affects binding or, in other words, that two tyrosine residues are needed for efficient binding to the substrate pocket.

### Limitations of the Study

The work was not carried out with the full SntA protein, nor with a cell-wall bound product, but with its soluble core, formed by the metallophos and the 5_nucleotid_C domains. The specificity and kinetics of the enzyme were studied only with Mn^2+^ as the activating cation. More research testing the effects of these variables will be required to obtain further insight into this interesting protein.

## Conclusion

SntA is a broad-specificity but selective phosphohydrolase. Taking as a reference the average value of catalytic efficiencies in the enzyme universe (≈ 10^5^ M^–1^ s^–1^) (Bar-Even et al., 2011), the repertoire of natural substrates that are hydrolyzed can be divided into two groups. Those with catalytic efficiencies (*k*_*cat*_/*K*_*M*_) ≥ 10^5^ M^–1^ s^–1^ include 3′-nucleotides, 2′,3′-cyclic nucleotides, and linear and cyclic dinucleotides and are considered as good substrates. Other compounds hydrolyzed by SntA with efficiencies <10^4^ M^–1^ s^–1^ are considered poor substrates.

The substrate repertoire of cell wall-bound SntA of *S. suis* is similar to that of periplasmic *E. coli* CpdB (López-Villamizar et al., 2016). Nevertheless, SntA is more efficient than CpdB with purine 3′-nucleotides, purine 2′,3′-cyclic mononucleotides, and linear or cyclic dinucleotides (all of them good SntA substrates with efficiencies ≥ 10^5^ M^–1^ s^–1^), SntA is, on the other hand, less efficient than CpdB with most of the poor SntA substrates and with the artificial phosphodiester bis-4NPhP. Therefore, SntA is much better adapted to hydrolyze its best natural substrates than CpdB, especially the cyclic dinucleotide c-di-AMP, for which SntA is 25-fold more efficient than CpdB.

The activity of SntA on c-di-AMP occurs with an efficiency similar to that shown by *S. agalactiae* CdnP. The hydrolysis of c-di-AMP by CdnP is instrumental in the evasion of the pathogen from the innate immune system of the infected host by extracellular hydrolysis of c-di-AMP, as this cyclic dinucleotide causes STING-dependent IFN induction (Andrade et al., 2016). This mechanism of virulence can also be operative in infections by *S. suis*, in addition to other reported effects of SntA as a mediator of iron acquisition and inhibitor of host AOP2 protein (Wan et al., 2017) or as a facilitator of complement evasion (Deng et al., 2018).

The substrate-binding site of SntA, located in the 5_nucleotid_C domain, contains two tyrosine residues (Y530 and Y633) that form a stacked sandwich with the purine base of substrates such as 3′-AMP. This interaction is important for substrate binding and, to a lesser extent, for substrate orientation toward the catalytic site in the metallophos domain. Both tyrosines are required for efficient binding of good SntA substrates, as reflected in the *K*_*M*_ values of mutant SntA proteins (Y530A, Y633A, and the double mutant Y530A + Y633A). For orientation toward the catalytic site, as reflected in *k*_*cat*_ values of the same mutants, one tyrosine is enough when 3′-AMP or 2′,3′-cAMP is the substrate, but this is not so with other good SntA substrates.

## Data Availability Statement

The supporting data may be requested from the corresponding authors without reservation.

## Author Contributions

JCC and JMR designed the study and wrote the manuscript. AC performed the molecular cloning of SntA and its core and constructed mutants. AC and MC expressed the recombinant proteins. MC, JC, and RP performed the kinetic assays. JCC analyzed these data. JRR and JMR performed bioinformatics and molecular modeling. All authors contributed to and revised the manuscript and approved the submission.

## Conflict of Interest

The authors declare that the research was conducted in the absence of any commercial or financial relationships that could be construed as a potential conflict of interest.

## Publisher’s Note

All claims expressed in this article are solely those of the authors and do not necessarily represent those of their affiliated organizations, or those of the publisher, the editors and the reviewers. Any product that may be evaluated in this article, or claim that may be made by its manufacturer, is not guaranteed or endorsed by the publisher.
